# Supplementary material for: Revised Annotations, Sex-Biased Expression, and Lineage-Specific Genes in the Drosophila melanogaster Group
Source: G3 (Bethesda). 2014 Oct 1;4(12):2345–51. doi: 10.1534/g3.114.013532 (PMC4267930; doi:10.1534/g3.114.013532)
Supplement: Supporting Information [file supp_4_12_2345__index.html]

Revised Annotations, Sex-Biased Expression, and Lineage-Specific Genes in the Drosophila melanogaster Group — Revised Annotations, Sex-Biased Expression, and Lineage-Specific Genes in the Drosophila melanogaster Group — Supporting Information 

# Revised Annotations, Sex-Biased Expression, and Lineage-Specific Genes in the *Drosophila melanogaster* Group

## Supporting Information for Rogers *et al.*, 2014

**Files in this Data Supplement:**

- Supporting Information - PDF, 660 KB
- File S1 - Data Archive (.zip, 17 MB)
