# Supplementary material for: Revised Annotations, Sex-Biased Expression, and Lineage-Specific Genes in the Drosophila melanogaster Group
Source: G3 (Bethesda). 2014 Oct 1;4(12):2345–51. doi: 10.1534/g3.114.013532 (PMC4267930; doi:10.1534/g3.114.013532)
Supplement: Supporting Information [file supp_g3.114.013532_013532SI.pdf]

G3: Genes | Genomes | Genetics

Revised annotations, sex-biased expression, and lineage-specific genes in the *Drosophila melanogaster* group

Rebekah L. Rogers[1], Ling Shao[1], Jaleal S. Sanjak [1], Peter Andolfatto[2], and Kevin R. Thornton[1]

1) Ecology and Evolutionary Biology, University of California, Irvine
2) Ecology and Evolutionary Biology and the Lewis Sigler Institute for Integrative Genomics, Princeton University

Corresponding author: Rebekah L. Rogers, Dept. of Ecology and Evolutionary Biology, 5323 McGaugh Hall, University of California, Irvine, CA 92697

Phone: 949-824-0614
Fax: 949-824-2181
Email: rogersrl@uci.edu

DOI: 10.1534/g3.114.013532

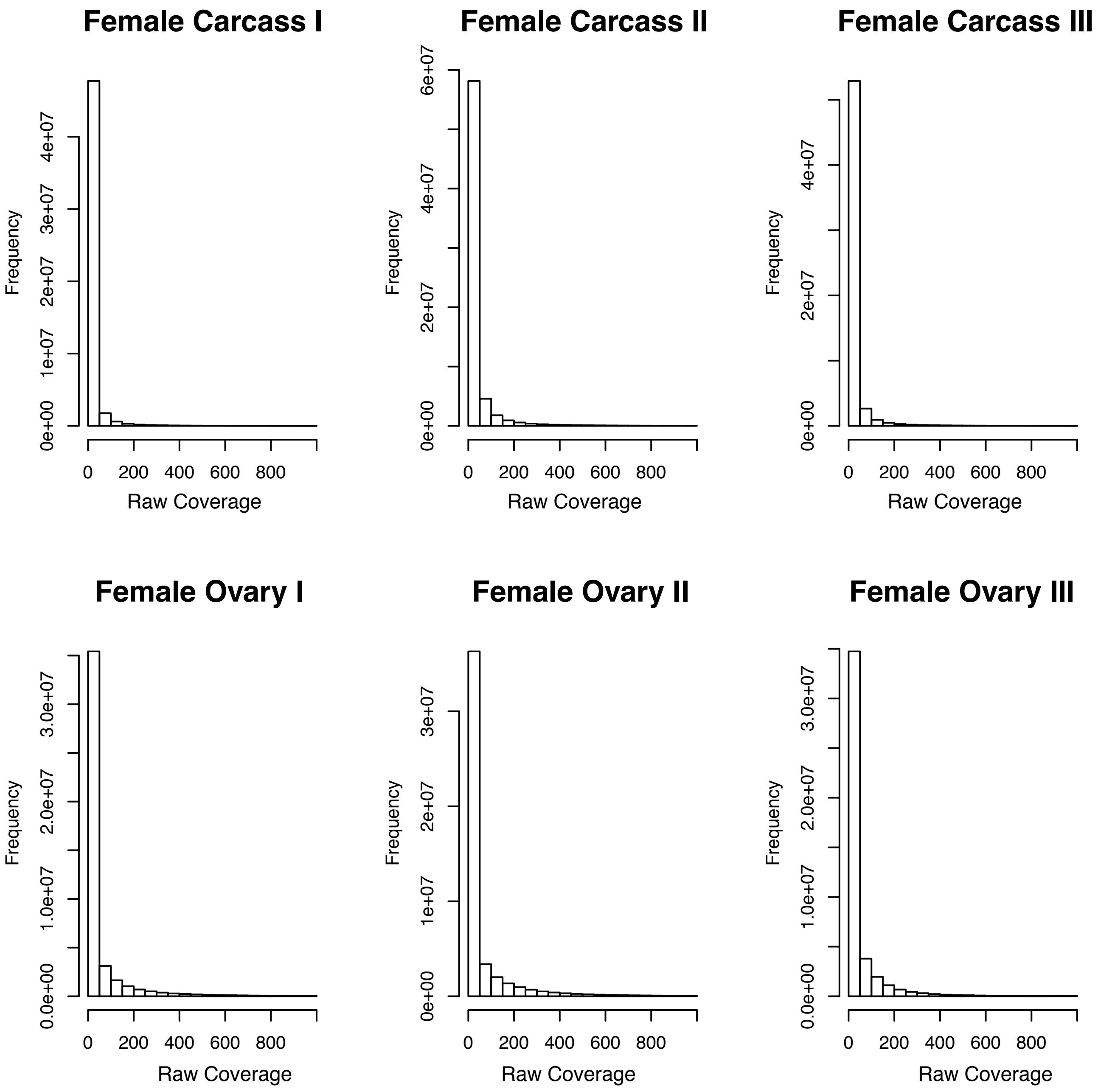

Figure S1: Raw coverage for sites with coverage sequencing depth between 1 and 1000 reads in RNA-seq data for replicates of female tissues in *D. simulans*.

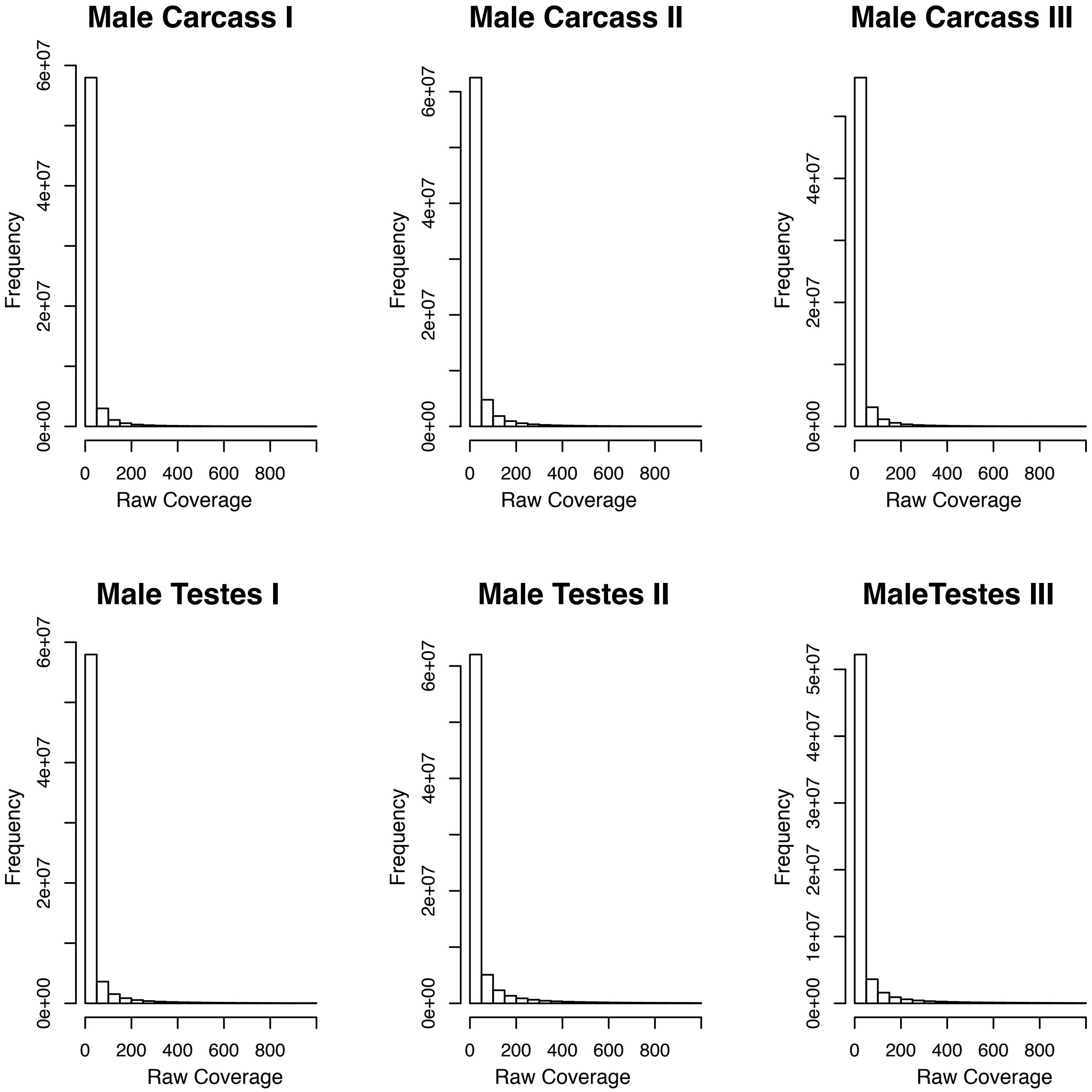

Figure S2: Raw coverage for sites with coverage sequencing depth between 1 and 1000 reads in RNA-seq data for replicates of male tissues in *D. simulans*.

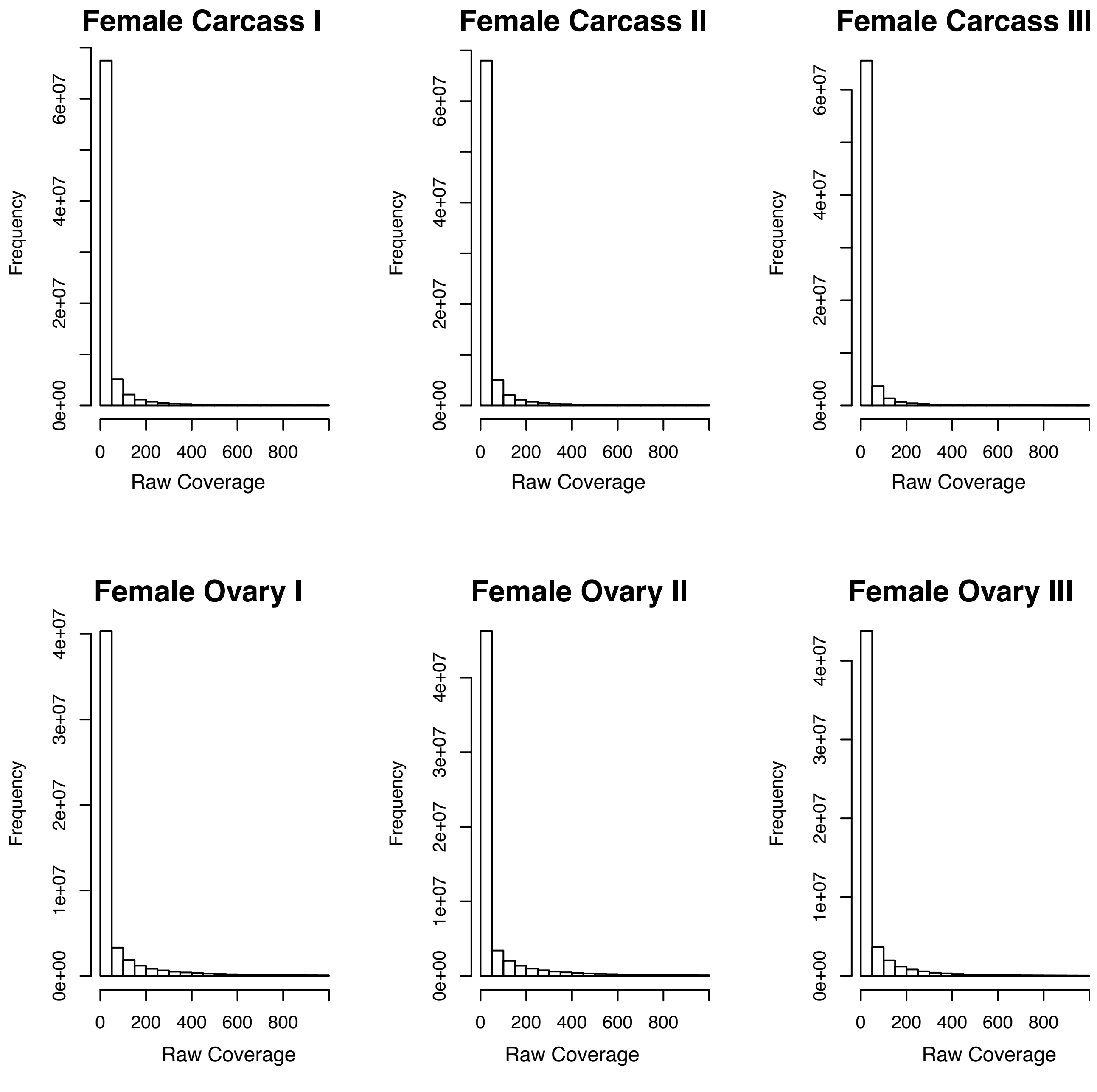

Figure S3: Raw coverage for sites with coverage sequencing depth between 1 and 1000 reads in RNA-seq data for replicates of female tissues in *D. yakuba*.

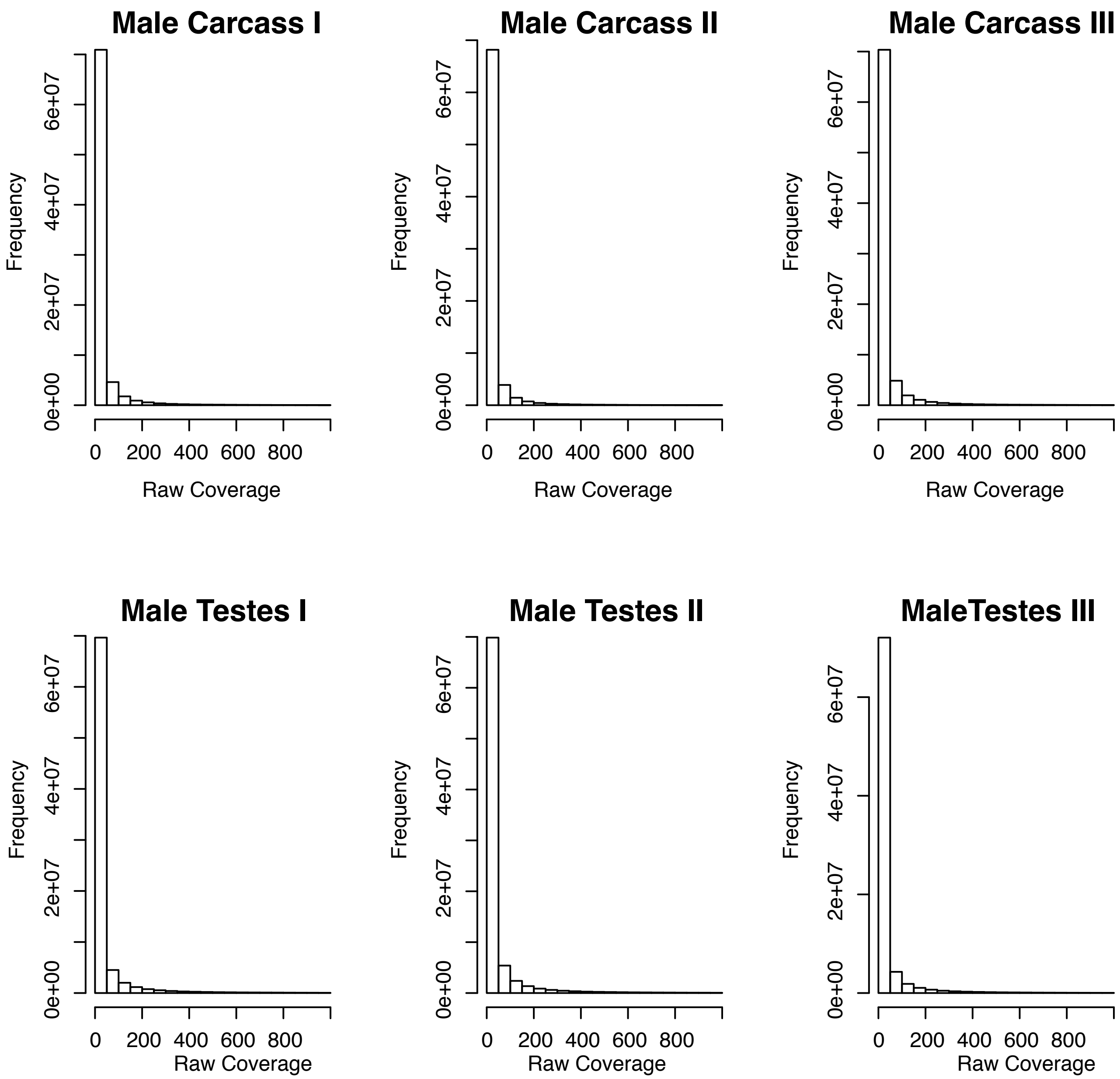

Figure S4: Raw coverage for sites with coverage sequencing depth between 1 and 1000 reads in RNA-seq data for replicates of male tissues in *D. simulans*.

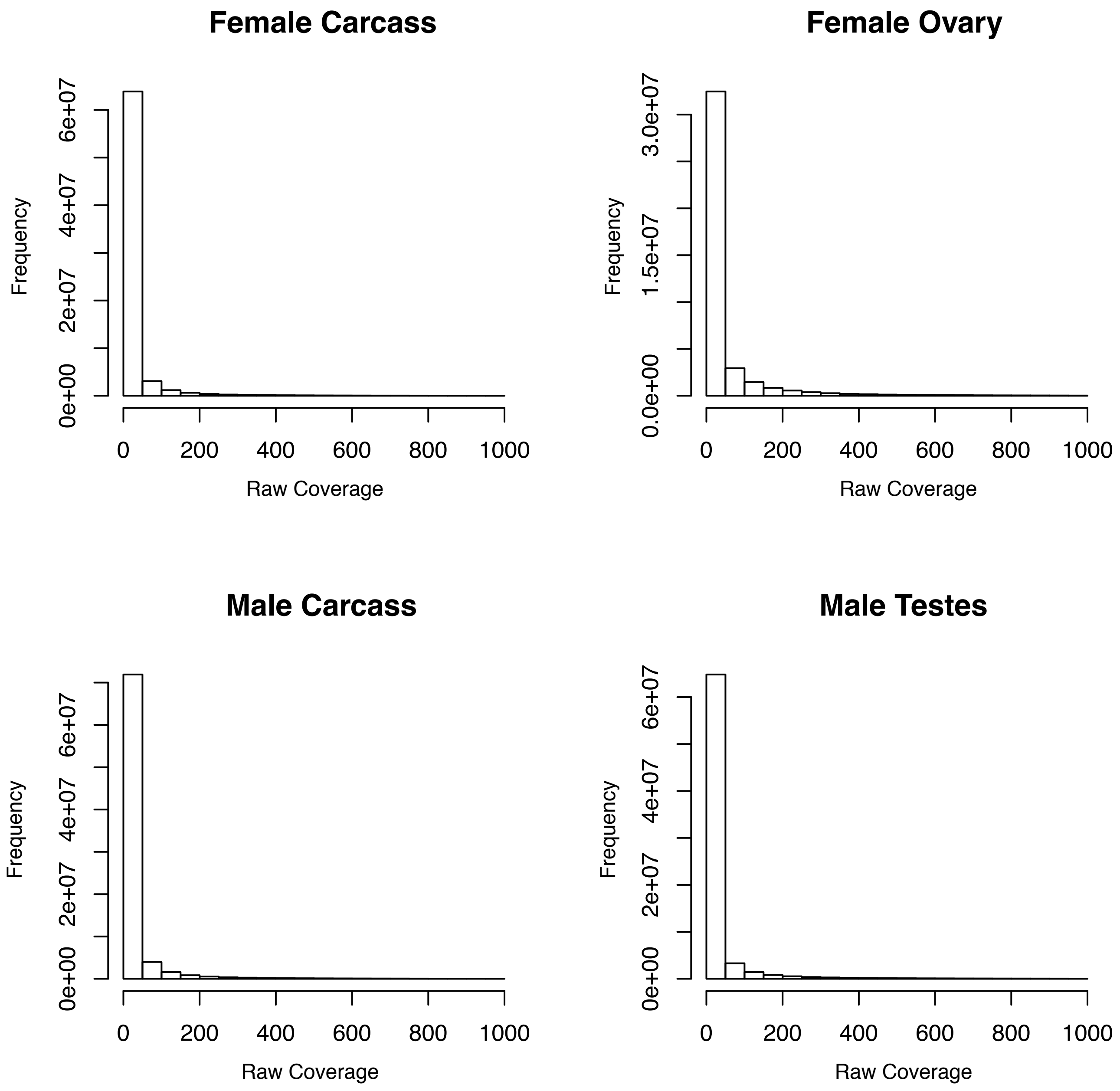

Figure S5: Raw coverage for sites with coverage sequencing depth between 1 and 1000 reads in RNA-seq data for tissues in *D. ananassae*.

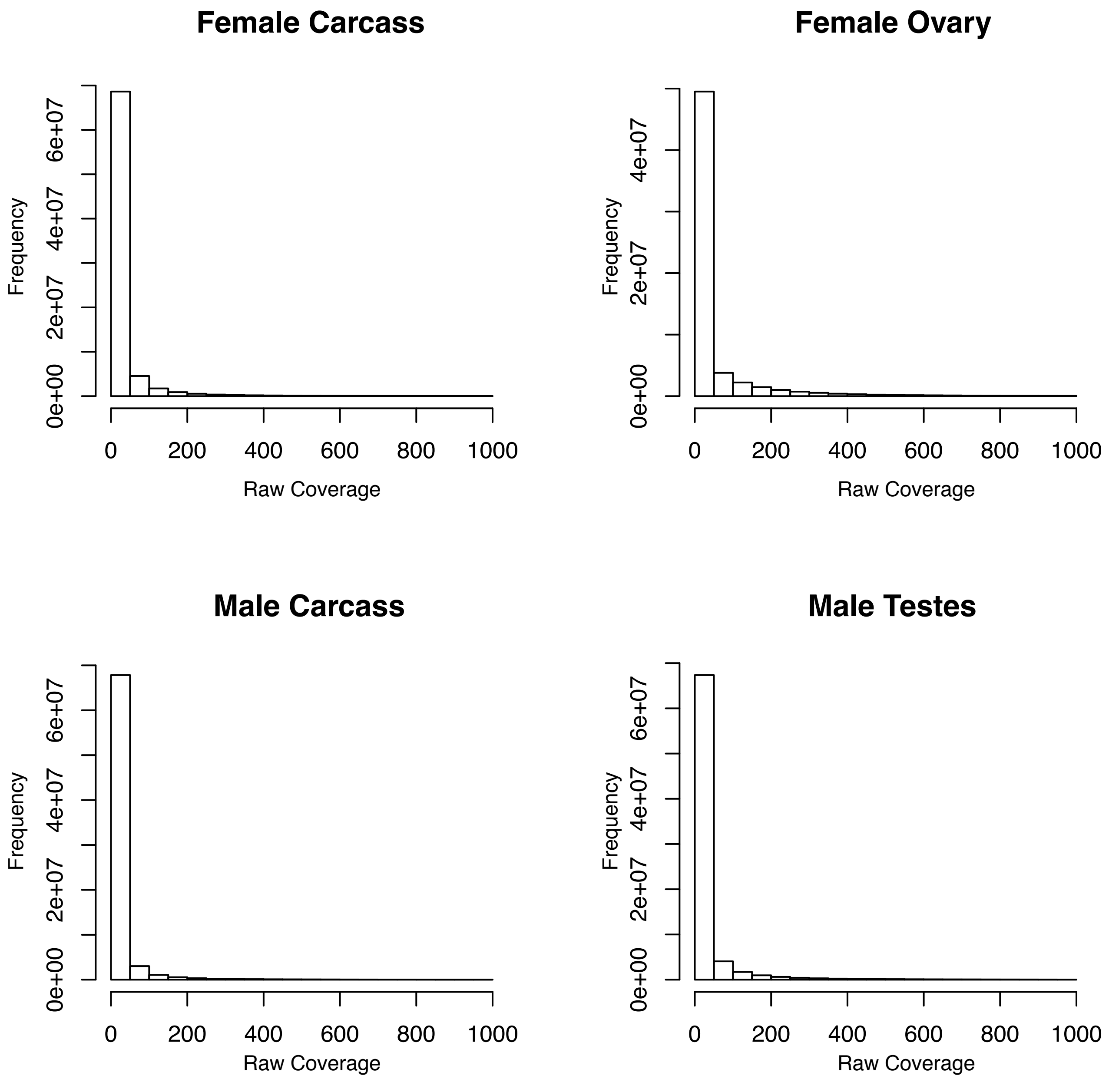

Figure S6: Raw coverage for sites with coverage sequencing depth between 1 and 1000 reads in RNA-seq data for tissues in *D. melanogaster*.

File S1

Data Archive

Available for download as a .zip file at http://www.g3journal.org/lookup/suppl/doi:10.1534/g3.114.013532/-/DC1
